# Quantifiable urine glyphosate levels detected in 99% of the French population, with higher values in men, in younger people, and in farmers

**DOI:** 10.1007/s11356-021-18110-0

**Published:** 2022-01-12

**Authors:** Daniel Grau, Nicole Grau, Quentin Gascuel, Christian Paroissin, Cécile Stratonovitch, Denis Lairon, Damien A. Devault, Julie Di Cristofaro

**Affiliations:** 1 Association Campagne Glyphosate, Foix, France; 2 Université de Pau Et Des Pays de L’Adour, CNRS, LMAP, E2S UPPA Pau, France; 3 ARSEAA, Pôle Guidance Infantile, Psychiatrie Infanto-juvénile Secteur III, Labège, France; 4 Faculté de Médecine de La Timone, Aix Marseille Université, INSERM, INRA, C2VN Marseille, France; 5 Centre Universitaire de Formation Et de Recherche de Mayotte, Dembeni, Mayotte France; 6 ADES, Aix Marseille University, CNRS, EFS, Marseille, France

**Keywords:** Glyphosate, France, General population, Dietary habits, Occupational exposure

## Abstract

France is the first pesticide-consuming country in Europe. Glyphosate is the most used pesticide worldwide and glyphosate is detected in the general population of industrialized countries, with higher levels found in farmers and children. Little data was available concerning exposure in France. Our objective was to determine glyphosate levels in the French general population and to search for an association with seasons, biological features, lifestyle status, dietary habits, and occupational exposure. This study includes 6848 participants recruited between 2018 and 2020. Associated data include age, gender, location, employment status, and dietary information. Glyphosate was quantified by a single laboratory in first-void urine samples using ELISA. Our results support a general contamination of the French population, with glyphosate quantifiable in 99.8% of urine samples with a mean of 1.19 ng/ml + / − 0.84 after adjustment to body mass index (BMI). We confirm higher glyphosate levels in men and children. Our results support glyphosate contamination through food and water intake, as lower glyphosate levels are associated with dominant organic food intake and filtered water. Higher occupational exposure is confirmed in farmers and farmers working in wine-growing environment. Thus, our present results show a general contamination of the French population with glyphosate, and further contribute to the description of a widespread contamination in industrialized countries.

## Introduction

Following World War II, the French agricultural model evolved through mechanization, crop improvement, and increased use of chemicals (plant protection products and fertilizers). Agriculture became more professional and specialized. In the last 40 years, the number of French farmers decreased from 1.61 million in 1982 to 0.4 million in 2019. Accordingly, 1.1 million farms were recorded in 1988 vs. 0.45 million in 2013. Nowadays, 29 million hectares (ha) are dedicated to agriculture (54% of the French surface area), with an average of 61 ha per farm. Farm sizes vary greatly according to crop production: 87 ha per farm for cereal production down to 10 ha for horticulture or market gardening (Agreste 2020b, d, e, INSEE 2020).

The use of pesticides resulted in the discharge of residues into the environment, ecosystem, and food chain (Hussain et al. 2015, Schulz et al. 2021). Despite the recent marked progression of organic production (Agreste 2020c), the purchase and use of pesticides in French agriculture increased by 25% during the last decade (Mandard 2020). France is among the world’s top ten pesticide-using countries (WorldAtlas 2021). In 2017, chemical weed control increased in almost all large-scale crops areas; data from the French Ministry of Agriculture indicate that the number of chemical sprays (herbicides, fungicides, pesticides) per crop range between 33 and 2.7, in decreasing order for fruit, wine, vegetable, and cereal farming (Agreste 2020a, e).

Glyphosate, put on the market in 1974 under the trade name “Roundup,” is the world’s most widely used broad-spectrum herbicide and crop desiccant, usually sprayed on weeds or some crops before harvest (Woodburn 2000). Glyphosate, or *N*-(phosphonomethyl) glycine, an organophosporus compound (phosphonate) (Franz 1974), blocks a metabolic pathway essential for the plant’s growth (Steinrucken and Amrhein 1980).

The glyphosate Maximum Residue Limit (MRL) in France for drinking water is 0.1 ng/ml. In solid food, MRL is higher and reaches 20 mg/Kg for cereals, like oats (20 mg/Kg), barley (20 mg/Kg), wheat (10 mg/Kg) or lentils (10 mg/Kg), beans (2 mg/Kg), peas (10 mg/Kg), and canola seeds (10 mg/Kg) (ANSES 2016, 2019). In France, glyphosate was found in 53% of food samples, including 87.5% of breakfast cereals; concentrations ranged from 40 μg/Kg for a breakfast cereal to 2100 μg/Kg for a sample of dry lentils (GénérationsFutures 2017). Another study conducted in France showed that glyphosate was found in 100% of infant cereal samples (ANSES 2016). In 2007, 9.5% of cereal samples tested in Europe by the European Food Safety Authority (EFSA) contained glyphosate. A study carried out in Switzerland on foods purchased in supermarkets found the highest levels of glyphosate in cereals and in pasta (Zoller et al. 2018). Glyphosate was also detected in beverages; in Germany, 6 out of 14 beers tested positive for glyphosate. All wines and fruit juices tested in Switzerland contained glyphosate (Zoller et al. 2018).

Human exposure to glyphosate, either by food and water intake or via external exposure, has been extensively studied. However, because of differences in methodology between studies, direct data comparison is difficult (Connolly et al. 2020a).

In the general population, the main route of exposure appears to be food, with higher levels of exposure in developing countries (Acquavella et al. 2004). Glyphosate was found in urine in the majority of studies (Gillezeau et al. 2019; Connolly et al. 2020a). Glyphosate was found in the urine of nearly half of the non-user volunteers from 18 European countries (IARC 2015). A literature review (Connolly et al. 2020a) reported that around 70% of urine samples were positive for glyphosate in the general population, with arithmetic mean concentrations varying between 0.28 ng/ml (McGuire et al. 2016) and 7.6 ng/ml (Varona et al. 2009). In Europe, a retrospective analysis of urine samples from Germany collected between 2001 and 2015 analyzed by GC–MS/MS (Gas Chromatography-Mass Spectrometry) reported glyphosate concentrations at or above the limit of quantification of 0.1 ng/ml in 31.8% samples, with a peak in 2012 (57.5%) and 2013 (56.4%) with median concentration slightly above the limit of quantification (LOQ) (Conrad et al. 2017). Connolly et al., validating a GC–MS protocol with a LOQ of 0.05 ng/ml, detected glyphosate in 66% of German urine samples (Connolly et al. 2020b). In a recent study conducted on Portuguese adults who mainly ate organic food, glyphosate measured in urine by GC–MS/MS was detected in 28% of samples (median value of 0.25 ng/ml) collected in July and detected in 73% of samples collected in October and analyzed by HPLC–MS/MS (high-performance liquid chromatography-mass spectrometry) (median value of 0.13 ng/ml) (Nova et al. 2020).

Men tend to have a higher mean urine concentration of glyphosate than women, and children a higher mean concentration than adults (Curwin et al. 2007; Conrad et al. 2017).

Recent European studies analyzed glyphosate levels in children; a study from Denmark showed that children presented higher glyphosate levels than their mother (Knudsen et al. 2017). This was confirmed by a German study reviewed in (Gillezeau et al. 2019); Lemke et al. (Lemke et al. 2021) showed that 52% of 2,144 first-void urine samples from German children and adolescents aged 3–17 years old had glyphosate level above the LOQ (0.1 ng/ml) with a geometric mean concentration of 0.107 ng/ml. Ferreira et al. (Ferreira et al. 2021) also detected glyphosate in 95.1% of 41 urine samples from Portuguese children (2–13 years old), with an arithmetic mean of 1.77 ng/ml and reaching a maximum value of 4.35 ng/ml. These authors reported values from previous studies conducted in children, with detection rates ranging from 11.1 to 100%, arithmetic mean values from 0.1 to 2.7 ng/ml, and maximum values from < 0.1 to 18 ng/ml (reviewed in (Ferreira et al. 2021)).

Pregnant women are also exposed to glyphosate (Parvez et al. 2018). In France, 43% of pregnant women had glyphosate in their urine (average 0.2 ng/ml and maximum 0.76 ng/ml) (Chevrier et al. 2009). An American multicenter survey observed glyphosate in 95% (LOD 0.014 ng/ml) of 2nd trimester maternal urine samples by UPLC-MS/MS (Ultra Performance Liquid Chromatography) with a median of 0.22 ng/ml (0.01 to 1.9 ng/ml) (Lesseur et al. 2021). In addition, Ruiz et al. (Ruiz et al. 2021) detected glyphosate in urine from 54% of Spanish breastfeeding mothers (*n* = 97) with a geometric mean of 0.12 ng/ml.

Occupational exposure occurs via the skin and via respiratory and digestive tracts, and is also valid for people living near agricultural holdings. Farmers and their families presented higher glyphosate levels than the general population; with glyphosate arithmetic mean levels reported in urine after work between 1.35 ng/ml in Europe (Connolly et al. 2017) and 292 ng/ml in China (Zhang et al. 2020). Glyphosate levels are also reported to be higher in farmers’ children (Jauhiainen et al. 1991; Curwin et al. 2007).

Important human health concerns have been raised regarding glyphosate exposure. The International Agency for Research on Cancer (IARC), a specialized agency of the World Health Organization (WHO), linked non-Hodgkin lymphoma (NHL) to glyphosate exposure and classified glyphosate as a “probable carcinogenic (Group 2 A)” (IARC 2015); this association has been further confirmed (Leon et al. 2019; Zhang et al. 2019; Inserm 2021), whereas the evaluation conducted by the European Food Safety Authority (EFSA) concluded that glyphosate is “unlikely to pose a carcinogenic hazard to humans and the evidence does not support classification with regard to its carcinogenic potential” (EFSA 2015). Evaluation by the EFSA mostly relied on studies conducted by agrochemical industries (Portier et al. 2016; Benbrook 2019; Foucart 2021a, b).

Some studies associated glyphosate herbicides to neurotoxic effects and impaired neurodevelopment (Nevison 2014; de Araujo et al. 2016; von Ehrenstein et al. 2019; Ongono et al. 2020); nephrotoxic mechanisms (Jayasumana et al. 2014; Gunarathna et al. 2018; Gunatilake et al. 2019); and endocrine disrupting effects, especially concerning sexual hormones (Savitz et al. 1997; Garry et al. 2002; Dallegrave et al. 2007; Alarcon et al. 2019; Manservisi et al. 2019; Ingaramo et al. 2020; Jarrell et al. 2020).

Due to the use of glyphosate in French agriculture, with available data indicating that glyphosate is frequently present in the food supply, we hypothesized that a large percentage of the French population would be contaminated by glyphosate.

Our aim in the present study was to evaluate the frequency and levels of glyphosate contamination in the French population, nationwide. We also aimed to determine a potential association of urine glyphosate levels with the seasons, subject characteristics, lifestyle status, dietary habits, or occupational exposure.

Based on the observation that measuring the concentration of glyphosate on a single urinary spot after an overnight fast is a reliable estimate of maximum glyphosate excretion in humans (Faniband et al. 2021), glyphosate was quantified in first-void urine samples from 6848 volunteer participants recruited throughout metropolitan France and in La Reunion Island (Indian Ocean).

## Material and methods

### Participants, sample, and data collection

The study was designed by the Campagne Glyphosate France Association (Foix, France). Participant recruitment was organized and conducted by French local committees between June 2018 and January 2020. One hundred and seventy-five sessions were organized in 63 French districts.

All subjects gave written informed consent to participate in the study prior to sample collection. No exclusion criteria were used to exclude potential participants.

A total of 6848 participants were recruited. Participants were asked not to urinate, drink, eat, or smoke at least 6 h before urine collection.

Prior to urine collection, a written questionnaire was filled in by each participant providing self-reported socio-demographic and lifestyle information. Collected data included age, gender, height and weight, employment status, place of residence, smoking status, physical activity practice, general dietary information (organic food consumption; beer and fruit juice consumption; tap, bottled, spring (well or natural source) or filtered water consumption). The questionnaire also included whether the participant had complied with the protocol before urine collection.

All samples were processed according a unique standardized protocol: anonymized first-void urine samples were collected in polypropylene centrifuge tubes (Nerbe Plus, Germany; #02–502-3001), incubated 10 min at 70 °C for stabilization and shipped at room temperature (RT) to Biocheck GmbH (Germany) for further analysis.

### Urine glyphosate quantification

Urine samples were analyzed for glyphosate residue levels using the glyphosate Enzyme-linked immunosorbent assays (ELISA) kit (Abraxis, Inc., USA; #500,086). The glyphosate ELISA kit quantification range in water on a direct sample is 0.075–4 ng/ml. Quantitative analytical method validation for water samples, performed by the Ontario Ministry of the Environment, showed that the correlation coefficient between LC–MS and ELISA was 0.804 (Abraxis Eurofin (Parmar)) and 0.88 according to another validation study (Byer et al. 2008). Quantification of glyphosate by ELISA in water samples evaluated according to HPLC showed a correlation of 0.99 (Clegg et al. 1999); similar performance of both methods were further confirmed (Rubio et al. 2003).

Urine samples were analyzed according to the manufacturer’s protocol, as validated by Krüger et al. (Krüger et al. 2014) based on ELISA and GC–MS assay data comparison on human urine samples.

All assays were performed by a single laboratory Biocheck GmbH. ELISA was performed according to the manufacturer’s protocol intended for human urine samples. Briefly, 500 µl of urine sample were filtered with a 3 K VWR Centrifugal filter at 3000 × *g* for 10 min and the upper layer was transferred to a new tube and analyzed according to the Glyphosate Plate ELISA Kit user’s guide. Samples with glyphosate concentrations higher than 4 ng/ml were diluted at a 1:5 ratio with the Glyphosate Sample Diluent provided in the kit and reanalyzed. Measurements were performed on a Sunrise Microplate Reader automate (Tecan, Switzerland). Standard deviation measured by intra-day and inter-day samples reported by Biocheck GmbH is 0.13 ng/ml.

### Statistical analyses and data interpretation

The participant data used in this study have been anonymized in the database. Glyphosate measurements and self-reported participant data on socio-demographic and lifestyle characteristics are reported as numbers, percentages or mean with range or standard deviation (SD). Geographical data are plotted on a map.

Glyphosate measurements were adjusted according body mass index (BMI, calculated as weight/height^2^) as proposed in (Boeniger et al. 1993). Differences in glyphosate concentrations according to each variable (age, gender, employment status, place of residence, smoking status, physical activity, dietary information) were assessed using ANOVA or Kruskal–Wallis tests.

All analyses were performed using R Environment for statistical computing (RCoreTeam 2020). The statistical significance level was set to *α* = 0.05.

## Results

### Population characteristics

**Fig. 1:**
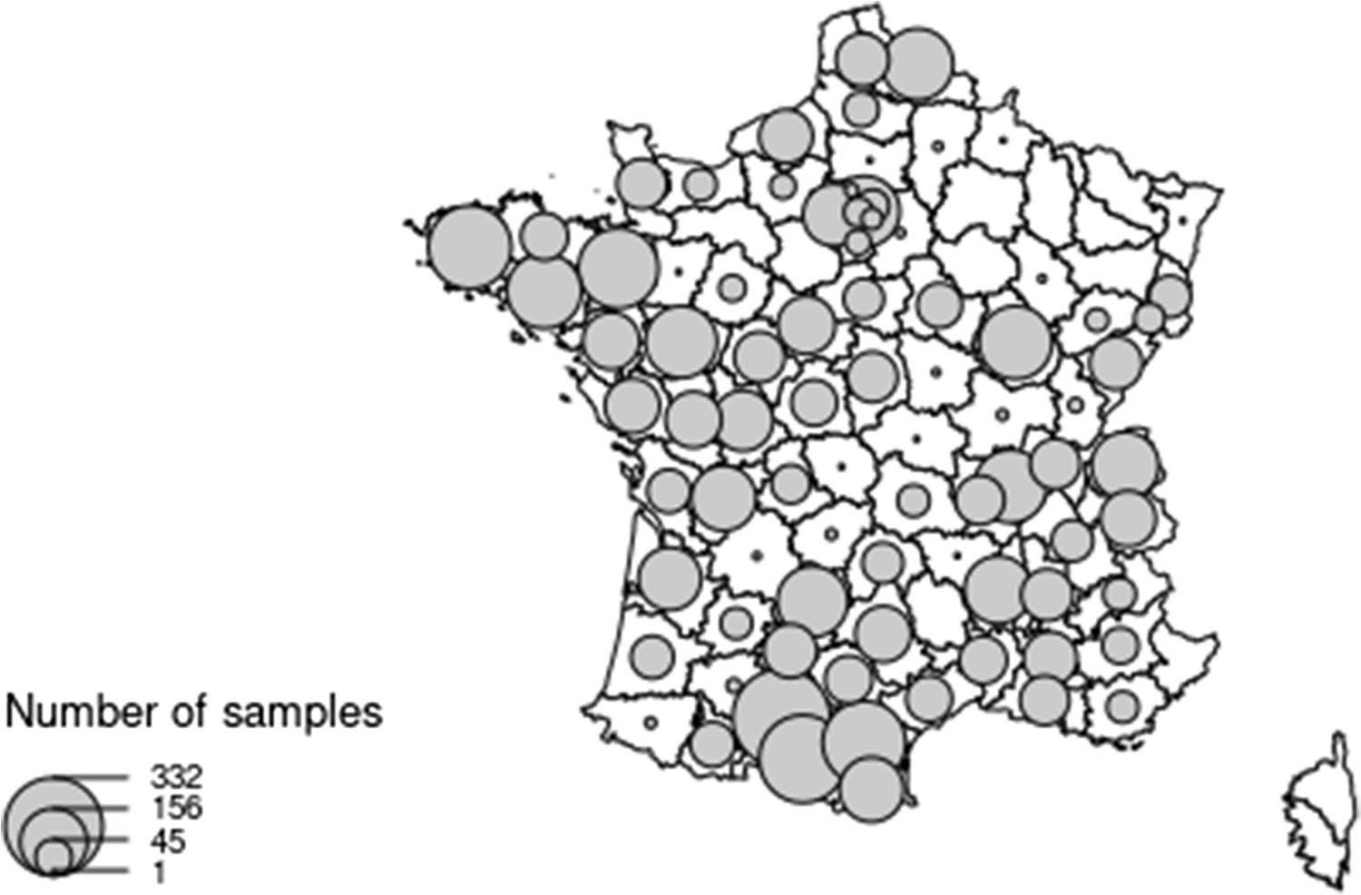
Geographical location of participants’ residence. Plot sizes are proportional to the number of participants

Among the 6848 urine samples collected, 53 (0.8%) could not be used and were excluded from further analysis; the cohort therefore included 6795 urine samples. Date and location of collection were available for all samples. Geographical location of participants’ place of residence is mapped in Fig. 1.

Five thousand eight hundred (5800) questionnaires filled in by these participants providing self-reported information were analyzed and further included in association analyses.

Participant characteristics are given in Table 1; 82% of participants self-reported compliance to the protocol, the median age was 53 years [0.5–94], and *M*/*F* sex ratio was 0.85. BMI, calculated according to height and weight, is presented in Table 1 according the WHO classification. Concerning lifestyle status, 11.0% of study participants self-reported no physical activity and 13.4% self-reported tobacco use. Concerning dietary habits, over half of the participants self-reported that their usual diet included at least 60% of organic food. Tap, bottled, spring (well or natural source) and filtered water, beer and fruit juice consumption were also reported in the questionnaire. Occupational status and working environment were also reported: notably, 6.3% of the participants were farmers and 28.6% were retired from the general population. A higher proportion of participants reported working in a countryside environment than in an urban environment; of note, 7.5% of the participants worked in a wine-growing environment.

**Table 1:** Participants’ self-reported biological, socio-demographic, and lifestyle information (*N* = 5800)

Characteristics	Study participants
Protocol compliance (%)	82.3
Age (years)	53 [0.5*–*94]
Gender (*M*/*F* ratio)	0.85
BMI, kg/m^2^ (%)	
Underweight, ≤ 18.5	7.0
Normal range, 18.5–24.9	67.4
Overweight, 25.0–29.9	20.7
Class I obesity, 30.0–34.9	3.8
Class II obesity, 35.0–39.9	0.8
Class III obesity, ≥ 40.0	0.2
Physical activity (%)	
Never	11.0
Occasionally	27.6
Regular basis	61.4
Smokers (%)	13.4
Proportion of organic food consumption (%)	
< 40%	17.3
40–60%	24.2
> 60%	58.4
Tap water consumption (%)	
Never or rarely	21.7
Occasionally	8.8
Every day or almost	69.5
Spring water consumption (%)	
Never or rarely	80.6
Occasionally	10.1
Every day or almost	9.2
Filtered water consumption (%)	
Never or rarely	73.0
Occasionally	4.3
Every day or almost	22.7
Beer consumption (%)	
Never or rarely	42.4
Occasionally	49.9
Every day or almost	7.7
Fruit juice consumption (%)	
Never or rarely	40.1
Occasionally	44.4
Every day or almost	15.5
Employment status (%)	
Retired	28.6
Farmers	6.3
Unemployed	5.8
Children	4.2
Other activity	55.1
Working environment* (%)	
Urban area	56.7
Countryside (excluding vineyards)	59.2
Vineyards	7.5

*Several answers could be given.

### Glyphosate is detected in 99.8% of the samples and is higher during spring and summer

Glyphosate was quantitatively detected in 6781 urine samples out of 6795 (99.8%). In association analysis, measurements below the LOQ (0.075 ng/ml) were considered to be equal to 0.0 ng/ml. BMI-adjusted glyphosate mean level was 1.19 ng/ml + / − 0.84, with a range [< 0.075; 7.36].

Data from the 5800 questionnaires were used to evaluate glyphosate levels according to the season of collection. Urine samples collected between May and September showed significantly higher glyphosate levels than those collected between October and April (Table 2; *p* < 0.001).

**Table 2:** BMI-adjusted glyphosate levels (ng/ml) according to seasons in all samples from France, and in samples collected in the same district at various seasons

	Spring–summer	Fall-winter
All samples (*N* = 5647)	1.40 + / − 0.93	1.05 + / − 0.74
Samples from same district (*N* = 1796)	1.43 + / − 0.88	1.05 + / − 0.75

Some urine collection sessions were repeated in the same geographical district: 1796 samples collected at least at 4 different times of the year in the same geographical district were available. Glyphosate measurements confirmed a higher level in spring–summer than in fall-winter (Table 2; *p* < 0.001).

### Glyphosate level is higher in first-void urine

Participants who reported to have urinated less than 6 h before urine collection (*N* = 983) displayed lower glyphosate level than participants who complied with the protocol (*N* = 4583) (0.95 ng/ml + / − 0.67 vs. 1.24 ng/ml + / − 0.83; *p* < 0.001).

### Glyphosate level is higher in men and in younger participants and decreases with age

Male participants (*N* = 2583) had higher mean glyphosate levels than women (*N* = 3040) (1.27 ng/ml + / − 0.84 vs. 1.13 ng/ml + / − 0.83; *p* < 0.001). Glyphosate levels were also higher in the youngest participants, with a continuous decrease with age (Table 3, *p* < 0.001).

**Table 3:** BMI-adjusted glyphosate level (ng/ml) according to participants’ age

Age (years)	Glyphosate level (ng/ml)
< 16 (*N* = 217)	2.05 + / − 1.29
16–39 (*N* = 1192)	1.44 + / − 0.92
40–49 (*N* = 1019)	1.26 + / − 0.83
50–59 (*N* = 1183)	1.11 + / − 0.73
60–69 (*N* = 1521)	0.99 + / − 0.67
70–79 (*N* = 468)	0.93 + / − 0.70
> 79 (*N* = 37)	0.67 + / − 0.58

### Glyphosate level is associated with smoking and dietary habits

#### Smoking is associated with higher glyphosate levels

Glyphosate level was higher in tobacco users (*N* = 717) than in non-smokers or former smokers (*N* = 4930) (1.43 ng/ml + / − 0.91 vs. 1.16 ng/ml + / − 0.82; *p* < 0.001).

#### Major organic food consumption is associated with lower glyphosate level

No statistically significant difference was observed between participants who consume organic food (any percentage) ( *N* = 5271) and those who do not consume any organic food (*N* = 216) (Table 4; 1.19 ng/ml + / − 0.84 vs. 1.17 ng/ml + / − 0.80; *p* = 0.68). However, participants who reported eating more than 85% of organic food (*N* = 1327) displayed lower glyphosate level than other participants (*N* = 3875) (1.16 ng/ml + / − 0.80 vs. 1.21 ng/ml + / − 0.85; *p* = 0.026).

**Table 4:** BMI-adjusted glyphosate level (ng/ml) according to participants’ organic food consumption

Organic food consumption	Glyphosate level (ng/ml)
No (*N* = 216)	1.17 + / − 0.80
Yes (*N* = 5271)	1.19 + / − 0.84
Yes, less than 85% (*N* = 3875)	1.21 + / − 0.85
Yes, more than 85% (*N* = 1327)	1.16 + / − 0.80

#### Beer and fruit juice consumption are associated with higher glyphosate level

Participants who drank beer had significantly higher glyphosate concentrations than other participants over 15 years old (Table 5; *p* < 0.001). Participants who drank fruit juice displayed higher glyphosate levels than non-consumers (Table 6; *p* = 0.009).

**Table 5:** BMI-adjusted glyphosate level (ng/ml) according to participants’ beer consumption

Beer consumption	Glyphosate level (ng/ml)
Never (*N* = 2153)	1.17 + / 0.87
Occasionally (*N* = 2542)	1.20 + / − 0.81
Regular basis (*N* = 378)	1.37 + / − 0.80

**Table 6:** BMI-adjusted glyphosate level (ng/ml) according to participants’ fruit juice consumption

Fruit juice consumption	Glyphosate level (ng/ml)
Never (*N* = 2151)	1.16 + / − 0.81
Occasionally (*N* = 2372)	1.21 + / − 0.86
Regular basis (*N* = 829)	1.25 + / − 0.85

### Tap and spring water consumption are associated with higher glyphosate levels whereas filtered water consumption is associated with lower glyphosate levels

Participants who drank tap water or spring water presented higher glyphosate levels (Table 7; *p* = 0.011 and Table 8; *p* = 0.025). Filtered water consumption was associated with lower glyphosate levels (Table 9; *p* < 0.001). Bottled water consumption was not associated with a change in glyphosate mean level (*p* = 0.83, data not shown). Most participants ticked several types of filter (including carbon filter, other filter, reverse osmosis, softener); thus, no association with a specific filter could be identified.

**Table 7:** BMI-adjusted glyphosate level (ng/ml) according to participants’ tap water consumption

Tap water consumption	Glyphosate level (ng/ml)
Never (*N* = 1208)	1.13 + / − 0.82
Occasionally (*N* = 492)	1.23 + / − 0.87
Regular basis (*N* = 3874)	1.20 + / − 0.83

**Table 8:** BMI-adjusted glyphosate level (ng/ml) according to participants’ spring water consumption

Spring water consumption	Glyphosate level (ng/ml)
Never (*N* = 4494)	1.18 + / − 0.83
Occasionally (*N* = 565)	1.27 + / − 0.87
Regular basis (*N* = 514)	1.23 + / − 0.90

**Table 9:** BMI-adjusted glyphosate level (ng/ml) according to participants’ filtered water consumption

Filtered water consumption	Glyphosate level (ng/ml)
Never (*N* = 4061)	1.21 + / − 0.84
Occasionally (*N* = 243)	1.31 + / − 0.94
Regular basis (*N* = 1268)	1.12 + / − 0.80

### Glyphosate level is associated to occupational exposure

Glyphosate levels were analyzed according to employment status. Farmers (*N* = 342) had significantly higher glyphosate concentrations than other participants over 15 years old (*N* = 4883) (1.29 ng/ml + / − 0.84 vs. 1.15 ng/ml + / − 0.79; *p* = 0.002).

When the specific work environment was considered, farmers working in a wine-growing environment (*N* = 63) presented higher glyphosate levels than other farmers (*N* = 279) (1.56 ng/ml + / − 0.98 vs. 1.22 ng/ml + / − 0.79; *p* = 0.004).

## Discussion

France is an important agricultural country, with half of its surface area dedicated to farming. During the last decades, the dominant French agricultural model evolved towards more intensive agriculture with an increased use of chemicals; France is among the top ten pesticide-consuming countries worldwide and is number one in the EU (Sharma et al. 2019).

Glyphosate has been extensively used since its commercialization in the 1970s in countries with intensive farming (reviewed in (Sharma et al. 2019)). Several methods have been developed and validated to measure glyphosate levels (reviewed in (Valle et al. 2019)). However, comparison of datasets needs to be carried out with caution, because of differences in sampling strategy, urinary dilution adjustments, or detection/quantification methods and limits (Connolly et al. 2020a). Accordingly, epidemiological studies on glyphosate have reported variable results. In Europe, the mean range of glyphosate levels was 0.16 to 7.6 ng/ml with detection frequencies in EU-states ranging between 10 and 90% (Conrad et al. 2017; Gillezeau et al. 2019; Connolly et al. 2020a; Nova et al. 2020). Little data is available concerning glyphosate levels in the French general population. A study conducted in an adult cohort on several pesticides (classified as organophosphorus, pyrethroid and azole compounds) showed lower exposure, based on urine sample levels, in frequent organic food consumers (Baudry et al. 2019).

The present study included 6848 participants recruited between June 2018 and January 2020. The whole of France was covered except the north-east area, and to a lesser extent, a corridor from the north east to the south west. Glyphosate was quantified in urine samples using ELISA assay by a single laboratory (*N* = 6795). Measuring glyphosate concentration on a single urinary spot early morning after an overnight fast has been assessed to be a reliable estimation of maximum glyphosate excretion in humans. Two experimental studies carried out on humans showed that urinary elimination of glyphosate followed a two-phase excretion, with an initial rapid phase between 6 and 9 h followed by a slower phase (Zoller et al. 2020; Faniband et al. 2021).

The ELISA method applied to glyphosate detection and quantification offers an alternative approach to the drawbacks of chromatographic techniques, such as the requirement of derivatization procedures, sample pre-treatments, costly equipment, and the speed of reactions and analysis. ELISA and HPLC methods show comparable performances in terms of accuracy and precision for the detection and quantification of glyphosate in water samples (Clegg et al. 1999; Rubio et al. 2003), as do ELISA and LC/MS methods in water (Byer et al. 2008). Although there is a strong correlation between ELISA and HPLC methods, Clegg et al. (Clegg et al. 1999) showed that glyphosate values determined by ELISA were greater than those obtained by the HPLC method. These results were confirmed in ELISA validation tests performed on 14 human urine samples; GC–MS and ELISA methods showed a correlation coefficient of 0.87 and mean values obtained with ELISA were higher (Krüger et al. 2014). Higher values of pesticides quantified by ELISA than by HPLC were also observed for atrazine mercapturate and chlorpyrifos (Curwin et al. 2010).

Thus, these methodological differences should be kept in mind concerning our results supporting a general contamination of the French population, with glyphosate quantifiable in 99.8% of urine samples and a mean of 1.19 ng/ml + / − 0.84.

Nevertheless, the biological, dietary habits and socio-demographic data association analyses performed here on a nationwide cohort, confirmed previously published data on glyphosate contamination.

Several studies performed with ELISA or LC methods found comparable results to ours in the general population in the USA, Denmark, or Sri Lanka (Curwin et al. 2007; Jayasumana et al. 2014; McGuire et al. 2016; Knudsen et al. 2017; Parvez et al. 2018; Lesseur et al. 2021), whereas other studies performed with GC or LC methods reported lower values in the USA, Germany, Portugal, or Spain (Mills et al. 2017; Connolly et al. 2018; Connolly et al. 2020a, b; Nova et al. 2020; Ruiz et al. 2021).

Compliance to the protocol (first-void urine collection) was associated with significantly higher glyphosate levels. These results support that delayed glyphosate excretion leads to higher urine concentrations. Some studies adjusted their results by a measurement of urinary dilution, mostly using creatinine. Experimental glyphosate intake followed by continuous assay monitoring recommended to adjust the urine dilution to obtain a better correlation (Zoller et al. 2020; Faniband et al. 2021); unadjusted and adjusted urinary excretion curves presented by Faniband et al. were very similar especially during the first 9 h (Faniband et al. 2021). However, creatinine is described to be affected by several factors as diet, age, sex, health status, including diabetes or kidney disorders. Other studies suggested to determine urine concentration by its specific gravity. All the tests used to measure urine specific gravity have certain limitations based on their underlying physical principles (Chadha et al. 2001). Since creatinine and specific gravity have drawbacks and an additional cost, concentration was adjusted according to BMI (Boeniger et al. 1993).

Our results seem to show a greater glyphosate intake in spring time. A former study showed that pesticides were more detected in groundwater during spring (McManus et al. 2014). Moreover, a Canadian study showed a bimodal glyphosate temporal distribution with peak concentrations occurring in late spring/early summer and fall (Byer et al. 2008). However, as no urine volume adjustment was performed and because people may excrete smaller volumes of urine during spring–summer than in winter, this result requires further investigation for confirmation.

Analysis of questionnaires revealed specificities of this participant cohort compared to the general population: older median age, more women, more physical activity, and less smokers (Galey et al. 2020, Pasquereau et al. 2020). Participants also displayed specific dietary habits with a greater organic food consumption (AgenceBio 2021). A high proportion of participants were farmers, including workers in a wine-growing environment (Cnav 2021). Recruitment was based on voluntary participation, which would explain such cohort specificities of citizens sensitized to pesticide issues and to living a healthy lifestyle.

Our results revealed an association between glyphosate levels and participant characteristics, as men presented higher glyphosate levels. Higher levels in men were previously reported (Conrad et al. 2017). Importantly, glyphosate also showed an inverse correlation with age, with the highest values in participants aged under 15, as previously reported (Curwin et al. 2007; Fagan et al. 2020). Higher glyphosate levels found in the youngest participants may be associated with dietary habits (especially infant cereal), physiology and metabolism (children breathe and drink twice more than adults), physical activities, behavior and hygiene patterns with higher soil ingestion (Moya et al. 2004; Ginsberg et al. 2016). The results presented here are comparable with those reported in Portugal (Ferreira et al. 2021) and in Denmark (Knudsen et al. 2017), both carried out by ELISA method and are higher than those reported in Germany (Lemke et al. 2021) or in the USA (Trasande et al. 2020).

Results according to dietary habits highlighted contamination by food intake as lower glyphosate levels were associated both with dominant consumption of organic food and of filtered water. No association with a specific filter could have be identified as most participants reported using several kinds of filter (including carbon filter, other filter, reverse osmosis, softener). Organic food consumption was previously shown to be associated with lower glyphosate levels (Fagan et al. 2020) or with lower levels of pesticides (Baudry et al. 2019). Both tap water and spring water consumption were associated with higher glyphosate values as compared to filtered water. Water contamination is common and likely as glyphosate is polar and water soluble. Monitoring pesticide residues in EU agricultural topsoil collected between 2015 and 2018 showed a maximum of 16 residues/sample with glyphosate being the most frequently detected and in highest contents (Geissen et al. 2021).

We also observed that higher glyphosate urine levels are associated with high beer and fruit juice consumption in concordance with studies showing noticeable glyphosate levels in beers and fruit juices (Zoller et al. 2018) and with reports showing that the number of chemical sprays per crop is the highest for fruit (Agreste 2020a, e).

Higher occupational exposure to pesticides was confirmed as farmers and more particularly farmers working in a wine-growing environment displayed higher glyphosate levels (respectively 1.29 ng/ml and 1.56 ng/ml). These results are supported by previously published data showing high levels in occupationally exposed groups, with mean values ranging from 1.35 to 3.2 ng/ml (Connolly et al. 2020a; Zhang et al. 2020). Two different studies performed with LC methods (Connolly et al. 2017, 2018) reported results that were comparable and lower than ours. The higher exposure levels found in farmers working in wine-growing may be due to a more intensive use of pesticides in vineyards (Agreste 2020a, e); in France in 2006, wine-growing represented 3.3% of agricultural land, whereas their pesticide consumption in euros was 14.4% (Butault et al. 2011). Furthermore, the use of plant protection products increased by 21% in wine-growing between 2010 and 2016 (Agreste 2019).

Thus, our present results on more than 6000 participants in various parts of the country firmly support a general contamination of the French population with glyphosate, with a significant seasonal effect backing contamination from external exposure. Our data confirm previous studies supporting higher levels in the young, in men and in occupationally exposed individuals. We also confirm glyphosate contamination via ingestion and inhalation, as lower levels of glyphosate were observed in individuals who mainly ate organic food and drank filtered water, while higher levels were found in tobacco users, glyphosate being used nowadays as a desiccant for some crops before harvest.

Our results are globally consistent with the data in international literature and show a large exposure of the French population to glyphosate-based herbicides. On the whole, this exposure seems comparable or slightly higher than that measured in inhabitants of other industrial countries.

It should be noted that our results do not allow to estimate the actual level of glyphosate daily intake in the population. The relevance of measuring the level of glyphosate in urine as a reliable estimate of exposure is a major methodological issue. Until recently, data were only available from laboratory animal studies, with excretion rates of approximately 20% of an orally administered dose of glyphosate (EFSA 2015). It was recently demonstrated, however, that about only 1% of the glyphosate dose was excreted in human urine within 44 h (Faniband et al. 2021). An accurate quantification of exposure, whether by internal or external route, is a major public health concern as toxicity evaluation relies on absorbed dose estimates (IARC 2015).

Our results concerning a higher contamination during spring and summer, along with a higher level found in non-filtered water consumers also raise the question of environmental contamination, supported by the widespread glyphosate contamination of honey (Zoller et al. 2018). Many studies associated pesticide use with the decline in numbers of insects and birds (Jactel et al. 2021). Moreover, resistance to glyphosate is a well-described mechanism (Sammons and Gaines 2014) that arises in several weed species, driven by rapid and different biological processes in response to abiotic selective pressure (Patterson et al. 2018, 2019). The fast and independent evolution in multiple species of resistance mechanisms to glyphosate makes it necessary to increase glyphosate applications to obtain an equivalent lethal effect. Accordingly, population exposure seems to have increased during the 2000s, as supported by a study carried out in Germany (Conrad et al. 2017).

In conclusion, our data further contribute to the description of a widespread glyphosate contamination of the population in industrialized countries and raise the question of the sustainability of widespread and repeated use of glyphosate. Glyphosate and pesticides, in general, are described as being harmful to both farmers’ health and biodiversity, with long-lasting environmental contamination. Although organic food production is continuously increasing in France (Agreste 2020c), glyphosate is still authorized in French and EU agriculture and the CAP (Common Agricultural Policy) recently adopted by European Union may not be strong enough to implement and support a transition towards a new agricultural model (Massot Marti 2020) rising to the challenge of food supply, farmers’ income and health and biodiversity.

## Data Availability

The datasets used and/or analyzed during the current study are available from the corresponding author on reasonable request.
